# Suppression of Intracellular Reactive Oxygen Species in Human Corneal Epithelial Cells via the Combination of Quercetin Nanoparticles and Epigallocatechin Gallate and In Situ Thermosensitive Gel Formulation for Ocular Drug Delivery

**DOI:** 10.3390/ph14070679

**Published:** 2021-07-15

**Authors:** Chuda Chittasupho, Taepin Junmahasathien, Jiratchaya Chalermmongkol, Raksakul Wongjirasakul, Phuriwat Leesawat, Siriporn Okonogi

**Affiliations:** 1 Department of Pharmaceutical Sciences, Faculty of Pharmacy, Chiang Mai University, Chiang Mai 50200, Thailand; jiratchaya_newzmine@outlook.com (J.C.); raksakul.sunsun@gmail.com (R.W.); phuriwat.l@cmu.ac.th (P.L.); siriporn.okonogi@cmu.ac.th (S.O.); 2 Research Center of Pharmaceutical Nanotechnology, Faculty of Pharmacy, Chiang Mai University, Chiang Mai 50200, Thailand

**Keywords:** ocular drug delivery, oxidative stress, quercetin, epigallocatechin gallate, cornea

## Abstract

Oxidative stress can cause several severe ophthalmological diseases. In this study, we developed a thermosensitive gel as a delivery system for two antioxidant substances, namely, quercetin and epigallocatechin gallate. The quercetin was loaded in the PLGA nanoparticles using a solvent displacement method. The physical and chemical stability of the quercetin nanoparticles were evaluated, and the degradation kinetics of the quercetin in the nanoparticles was investigated. The in vitro antioxidant and intracellular reactive oxygen species inhibition of the quercetin nanoparticles, combined with the epigallocatechin gallate (EGCG), were determined using a 2,2-diphenyl-1-picrylhydrazyl radical scavenging assay and a 2,7-dichlorodihydrofluorescein fluorescent probes, respectively. The thermosensitive gel loaded with the quercetin nanoparticles and EGCG was formulated. We confirmed that quercetin nanoparticles displayed the desired physical characteristics, release kinetics, and stability. The combination of quercetin nanoparticles and EGCG suggested the additive effect of antioxidant activity. We also demonstrated the superior intracellular ROS inhibition activity of the quercetin nanoparticles and EGCG with n-acetyl cysteine. The thermosensitive gel showed an appropriate gelation temperature and time for ocular drug delivery. Our results provide promising prospects for applying the thermosensitive gel loaded with quercetin nanoparticles and EGCG as an efficient drug delivery system for antioxidant activity in human corneal epithelial cells.

## 1. Introduction

Oxidative stress results from an imbalance between reactive oxygen species (ROS) production and the ability of antioxidant ROS scavenging systems. The increased intracellular levels of ROS are associated with cellular and tissue damage, including oxidizing DNA, proteins, and lipid membranes [1]. Oxidative stress can cause several ophthalmological diseases and disorders, including age-related macular degeneration, cataract, uveitis, diabetic retinopathy, glaucoma, and inflammation [2,3,4,5,6,7,8,9,10]. The eyes’ cornea is a barrier against eye injury and trauma caused by the Sun’s rays, UV radiation, and pollutants. It is composed of the outer epithelium, corneal fibroblasts, and the inner endothelium. The cornea contains natural antioxidants that remove free radicals and ROS, namely, non-enzymatic antioxidants, such as vitamin C, α-tocopherol, and β-carotene, as well as enzymatic antioxidants, such as superoxide dismutase, catalase, glutathione reductase, and aldehyde dehydrogenase [11]. However, the continuous exposure of the cornea to oxidative stress can lead to the accumulation of ROS and oxidative stress generation. Oxidative stress and aging can reduce the level of antioxidants and the activity of antioxidant enzymes, leading to functional and structural changes of corneal epithelial cells, fibroblasts, and endothelial cells [12].

Quercetin is a natural compound that is classified as a polyphenolic flavonoid. Quercetin has shown a strong antioxidant activity by scavenging free radicals, reducing ROS levels, enhancing the expression levels of endogenous antioxidant enzymes, and modulating antioxidant enzyme-related genes, both in vitro and in vivo [13]. Quercetin inhibits oxidative stress and prevents oxidative damage. It was shown to protect cells from genetic toxicity and damage induced by radiation via free radical scavenging and increasing endogenous antioxidant enzyme levels. The limitation of quercetin use is due to its poor water solubility and low bioavailability (5.3%) [14]. Nanoparticle-based drug delivery systems were exploited to improve the solubility and bioavailability of quercetin. Chen et al. reported that a liposomal copper (II)-quercetin formulation can increase the apparent solubility of quercetin more than 100-fold [15]. Jain et al. presented a quercetin-loaded self-nanoemulsifying drug delivery system (SNEDDS) and showed that quercetin-loaded SNEDDS can enhance antioxidant and anti-cancer efficacy compared with free quercetin [16]. Quercetin Phytosome^®^, a lecithin formulation, significantly improved in vitro solubility and oral absorption in healthy volunteers [17]. Quercetin-loaded polymeric micelles significantly improved the relative oral bioavailability by 286% compared with free quercetin and extended the half-life of quercetin to 2.19-fold longer [18].

Topical ophthalmic formulations, such as solutions, suspensions, and ointments, are commonly used to deliver the drugs to an anterior segment of the eyes. Topical ocular drug delivery has advantages over other routes of administration due to its non-invasiveness, low systemic side effects, avoiding the first-pass metabolism, and ease of administration [19]. The bioavailability of ophthalmic drugs can be enhanced by prolonging corneal residence time and enhancing drug penetration. Nanoparticles (NPs) have advantages over conventional ophthalmic formulations by extending the drug retention time on the cornea, providing sustained release of the drug, increasing drug permeability and solubility, reducing drug degradation from enzymatic activity, and reducing the frequency of dose [20]. The goal of this study was to develop and analyze the physical and chemical characteristics of the quercetin-loaded PLGA NPs and in situ thermosensitive gel. The in vitro and intracellular antioxidant activity of quercetin-PLGA NPs in combination with EGCG were investigated. The cytotoxicity of the quercetin-loaded PLGA NPs combined with EGCG against human cornea epithelial cells was determined. An in situ thermosensitive gel containing quercetin-loaded PLGA NPs combined with EGCG was developed for an ocular drug delivery system.

## 2. Results

### 2.1. Preparation and Characterization of the Quercetin-Loaded PLGA NPs

**Figure 1:**
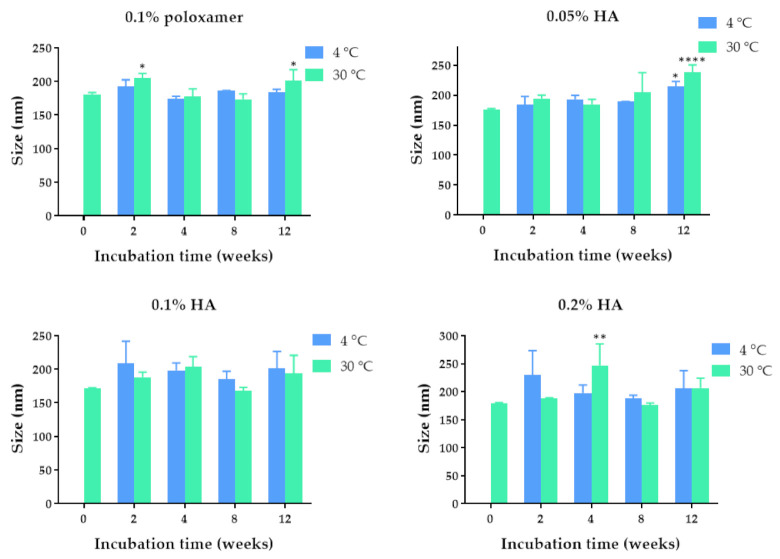
The hydrodynamic diameter of the quercetin-loaded PLGA NPs after fresh preparation and storage in deionized water for 2, 4, 8, and 12 weeks at 4 °C and 30 °C, respectively. *, **, and **** indicate *p* < 0.05, 0.01, and 0.0001 compared with day 0, respectively.

**Figure 2:**
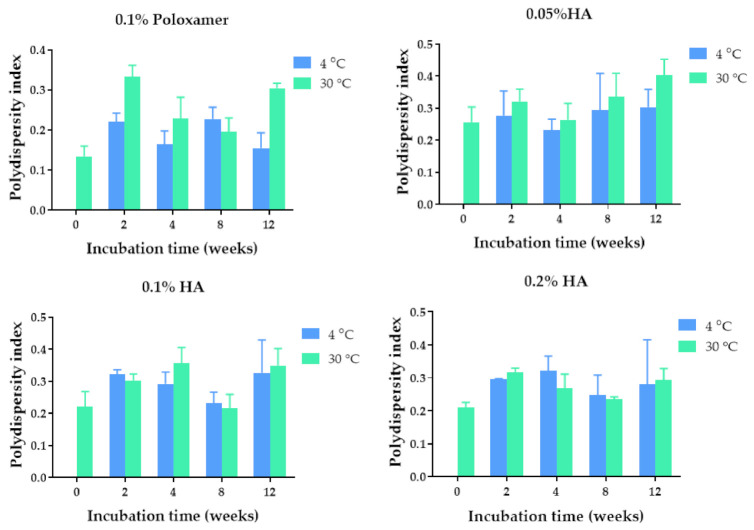
Polydispersity index of the quercetin-loaded PLGA NPs after fresh preparation and storage in deionized water for 2, 4, 8, and 12 weeks at 4 °C and 30 °C.

**Figure 3:**
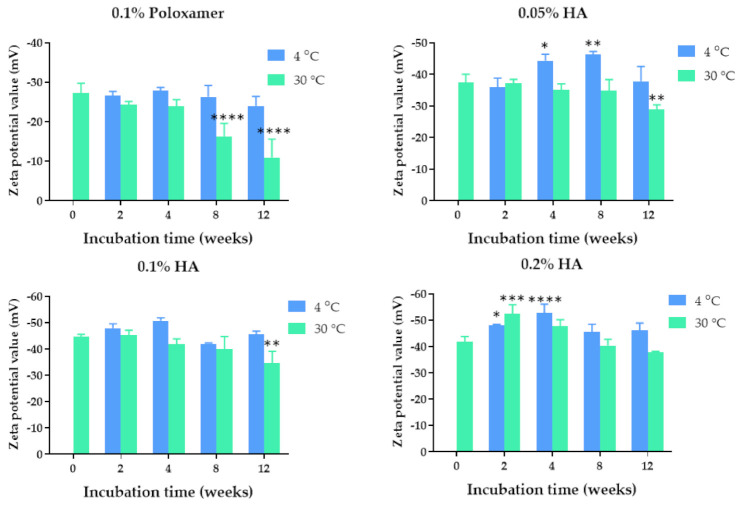
Zeta potential values of the quercetin-loaded PLGA NPs after fresh preparation and storage in deionized water for 2, 4, 8, and 12 weeks at 4 °C and 30 °C. *, **, *** and **** indicate *p* < 0.05, 0.01, 0.001, and 0.0001 compared with day 0, respectively.

The quercetin-loaded PLGA nanoparticles were developed by using the solvent displacement method. Poloxamer 407 (0.1%) and hyaluronic acid (0.05%, 0.1%, and 0.2%) solutions were used as stabilizers based on the biocompatibility with corneal tissue [21,22]. The colloidal characteristics of the quercetin-loaded PLGA NPs coated with poloxamer and HA, including size, polydispersity index, and zeta potential value of the nanoparticles, are shown in Figure 1, Figure 2 and Figure 3. The quercetin-loaded PLGA NPs that were formulated by using 0.1% poloxamer 407 as a surfactant had the lowest polydispersity index and the lowest negative charge. The quercetin-loaded PLGA NPs that were developed by utilizing 0.1% HA as a stabilizer were the smallest, had an acceptable polydispersity index, and had the highest negative surface charge. The negative zeta potential value was due to the free carboxylic groups of hyaluronic acid that coated the quercetin-loaded PLGA NPs. The NPs with a highly negative zeta potential value were expected to be colloidal stable due to the repulsive force and hydrophilic steric hindrance between nanoparticles. Therefore, this formulation was selected for further studies.

### 2.2. Physical Stability of the Quercetin-Loaded PLGA NPs in Aqueous Solution

The physical stability of the quercetin-loaded PLGA NPs in an aqueous solution was examined by measuring the size, polydispersity index, and zeta potential values of the quercetin-loaded PLGA NPs stored at 4 °C and 30 °C for 2 weeks and 1, 2, and 3 months. Among all the formulations, the quercetin-loaded PLGA NPs that were stabilized by using 0.1% HA were mostly stable when stored at 4 °C and 30 °C. The maximum hydrodynamic diameter was 209 nm and 204 nm after storage at 4 °C and 30 °C, respectively (Figure 1). The sizes of the quercetin-loaded NPs coated with 0.1% HA stored at 4 °C and 30 °C for 3 months were not significantly different compared with that of freshly prepared NPs, suggesting the physical stability of the quercetin-loaded PLGA NPs. The polydispersity indexes of the quercetin-loaded PLGA NPs stabilized with 0.1% HA ranged from 0.222–0.350 when they were stored at 4 °C and 30 °C for 3 months, indicating a narrow size distribution. It was found that all the quercetin-loaded PLGA NPs coated with different types and concentrations of stabilizers did not have any significant changes in polydispersity index during storage at 4 °C and 30 °C for 3 months, suggesting the physical stability of quercetin-loaded NPs from surface modification and steric stabilization (Figure 2) [23]. The quercetin-loaded PLGA NPs coated with 0.1% HA had significantly decreased negative zeta potential values when the NPs were stored at 30 °C for 3 months (Figure 3). The zeta potential of the quercetin-loaded NPs did not change after storage at 4 °C for 3 months. A more negative zeta potential value may result in higher colloidal stability of the nanoparticles due to the electrostatic interaction. The quercetin-loaded PLGA NPs stabilized with 0.1% poloxamer 407, 0.05% HA, and 0.2% HA were not as stable as the quercetin-loaded NPs coated with 0.1% HA. Therefore 0.1% HA quercetin-loaded PLGA NPs were further investigated for other characteristics.

### 2.3. Colloidal Stability of the Quercetin-Loaded PLGA NPs in PBS

**Figure 4:**
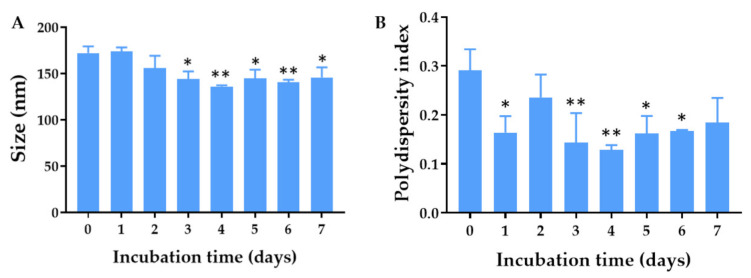
(**A**) Hydrodynamic diameter and (**B**) polydispersity index of the quercetin-loaded PLGA NPs after fresh preparation and storage in phosphate buffer saline for 1, 2, 3, 4, 5, 6, and 7 days at 37 °C. * and ** indicate *p* < 0.05 and 0.01 compared with day 0, respectively.

The ability of the quercetin-loaded PLGA NPs to retain their stability in a corneal environment was investigated by evaluating the colloidal stability of the NPs in Dulbecco’s phosphate buffer saline, which was used as a simulated tear fluid [24]. The results showed no apparent increase in the nanoparticle size, demonstrating colloidal stability of the NPs under the simulated physiological condition (Figure 4). In contrast, the size and size distribution were decreased, which might have been due to the release of quercetin and the electrostatic repulsion with components of the PBS, respectively. The stability of the NPs in PBS could be attributed to the presence of a hyaluronic acid coating on the NPs’ surfaces. Hyaluronic acid adsorbed on the surface of PLGA NPs was shown to provide steric hindrance and prevent PLGA NPs from aggregation and precipitation [25].

### 2.4. Encapsulation Efficiency and Release of Quercetin from the Quercetin-Loaded PLGA NPs

**Figure 5:**
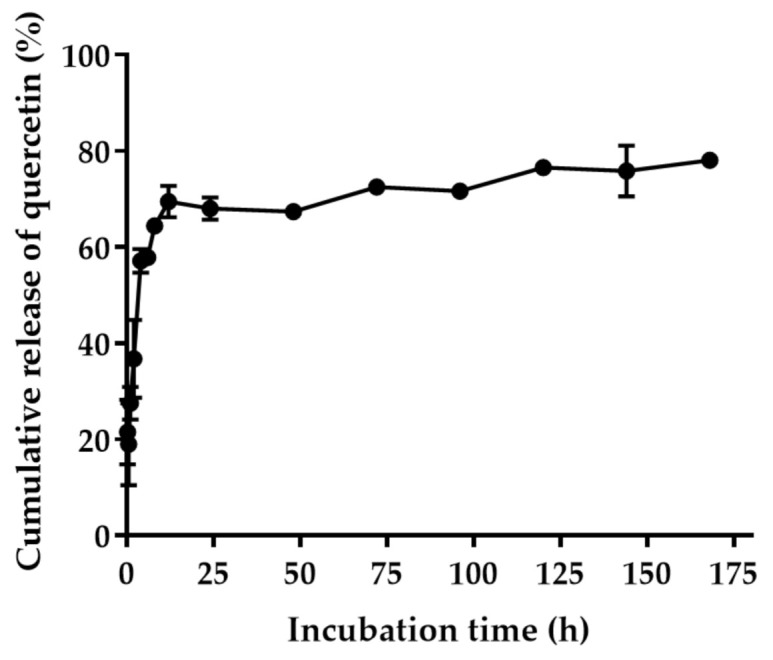
The cumulative release of quercetin from the quercetin-loaded PLGA NPs in phosphate buffer saline, pH 7.4, at 37 °C.

The encapsulation efficiency study showed that 93.83 ± 6.27% of the quercetin was entrapped in the 0.1% HA PLGA NPs. The loading efficiency of quercetin in PLGA nanoparticles was 10.54 ± 2.34%. The cumulative percent release of quercetin is shown in Figure 5. The burst release of quercetin was observed for the first 12 h of incubation in PBS at 37 °C, which might have resulted from the release of quercetin adsorbed on the surface of the NPs. After 12 h, the quercetin was released from the PLGA NPs via diffusion of the aqueous medium into the polymer matrix, which was brought about by the diffusion of the encapsulated quercetin toward the bulk aqueous medium [26]. Therefore, the quercetin released from PLGA NPs was matrix-dependent and depended on the erosion rate of the polymer and allowed for the partitioning of quercetin into the aqueous medium. The developed PLGA nanoparticles could improve the solubility of quercetin and sustained the release of quercetin.

### 2.5. Chemical Stability of Quercetin in the PLGA NPs

**Figure 6:**
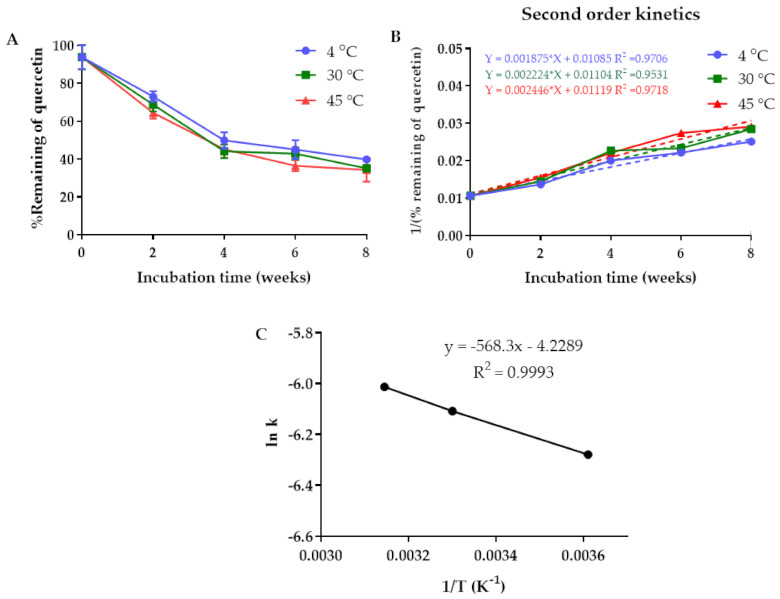
(**A**) Percentage remaining of the quercetin in PLGA NPs after storage at 4 °C, 30 °C, and 45 °C for 8 weeks. (**B**) Second-order kinetics model of the quercetin degradation. (**C**) Arrhenius plot for the quercetin in PLGA NPs degradation.

The percentage remaining of quercetin in the PLGA nanoparticles decreased over the storage time. The degradation rate constant of quercetin (k) was time- and temperature-dependent. There were 40%, 35%, and 34% of the quercetin remaining in the NPs after storage at 4 °C, 30 °C, and 45 °C for 8 weeks, respectively (Figure 6A). The kinetic degradation of quercetin followed a second-order reaction as a plot of 1/(percentage remaining of quercetin) against time yielded straight lines with the correlation coefficients of 0.9706, 0.9531, and 0.9718 for kinetic degradation at 4 °C, 30 °C, and 45 °C, respectively (Figure 6B). According to the second-order kinetics, the half-lives of quercetin loaded in the NPs were 5.3, 4.5, and 4.1 weeks when stored at 4, 30, and 45 °C, respectively. The Arrhenius plot revealed that the rate constant of the reaction increased with an increasing storage temperature (Figure 6C). The results suggested that quercetin-loaded NPs should be stored at 4 °C to prolong their chemical stability.

### 2.6. Antioxidant Activity of Quercetin-Loaded NPs Mixed with EGCG

**Figure 7:**
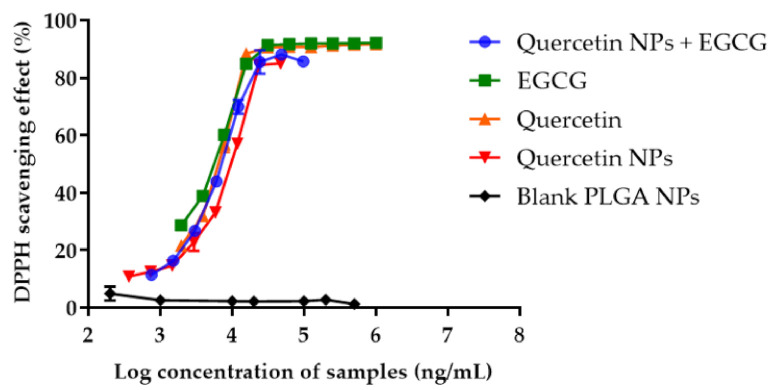
Percentages of the radical scavenging activity of pure quercetin, pure EGCG, quercetin-loaded NPs, quercetin- loaded PLGA NPs combined with EGCG, and blank PLGA NPs, which were determined using the DPPH free radical scavenging method.

The antioxidant activity of the combination of the quercetin-loaded PLGA NPs and EGCG was studied by investigating the free radical scavenging assay. The result was compared with the antioxidant activities of pure quercetin, pure EGCG, the quercetin-loaded PLGA NPs, the quercetin-loaded PLGA NPs combined with EGCG, and blank PLGA nanoparticles. The antioxidant activities of all samples, except the blank PLGA NPs, were concentration-dependent (Figure 7). The IC_50_ values for pure EGCG, pure quercetin, quercetin in PLGA NPs combined with EGCG, and quercetin in PLGA NPs were 4.99 ± 0.13 µg/mL, 5.89 ± 0.25 µg/mL, 6.71 ± 0.32 µg/mL, and 8.45 ± 0.23 µg/mL, respectively. Blank PLGA NPs did not show any antioxidant effect. The free radical scavenging activity of the quercetin-loaded PLGA NPs combined with EGCG was 1.26-fold higher than that of the quercetin in PLGA NPs alone. These results suggested that the combination of the quercetin-loaded PLGA NPs with EGCG had an additive effect on the antioxidant activity. The lower antioxidant activity of quercetin encapsulated in the PLGA NPs compared with pure quercetin resulted from the slow release of quercetin into the medium.

### 2.7. Effects of the Quercetin-Loaded PLGA NPs Combined with EGCG on HCE Cell Viability

**Figure 8:**
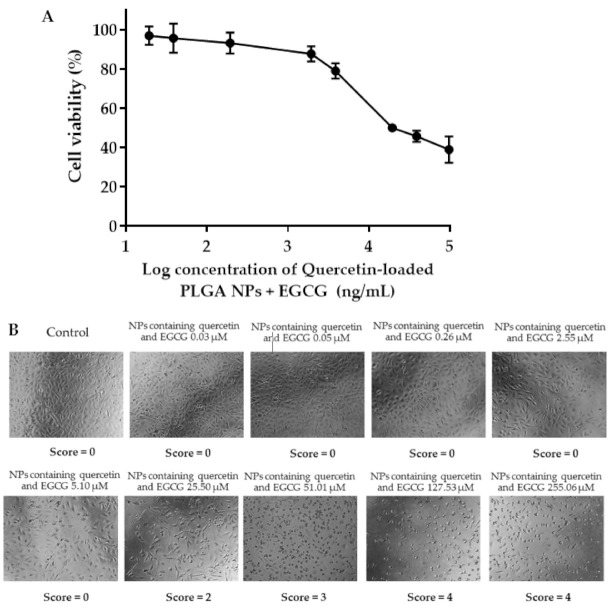
(**A**) Effect of quercetin-loaded PLGA NPs combined with EGCG on the HCE cell viability after incubation for 24 h. (**B**) Changes in the morphology and density of the HCE cells after 24 h of exposure to various concentrations of quercetin-loaded PLGA NPs and EGCG.

The cytotoxicity of quercetin-loaded in PLGA NPs combined with EGCG was evaluated in the HCE cell line. The cells were exposed to quercetin-loaded NPs combined with EGCG in the concentration range of 0.02–194 µg/mL quercetin and EGCG equivalent. The increase in the concentration of quercetin-loaded PLGA NPs and EGCG decreased the cell metabolic activity of the HCE cell line in a dose-dependent manner (Figure 8). The reduction in the HCE cell metabolic activity suggested an increase in cell death. The IC_50_ value of quercetin loaded in NPs and EGCG against the HCE cell line was 48.24 µM. The reduction in cell viability after exposure to a high concentration of quercetin-loaded NPs combined with EGCG was confirmed by observing the HCE cell morphology and cell density. The results showed that some cells that were exposed to quercetin in the NPs and EGCG at concentrations of 25.5–255.1 µM had a spherical shape compared with the spindle shape of normal cells. In addition, there was a decrease in a number of cells after the treatment, with a higher concentration of the quercetin loaded in PLGA NPs and EGCG. These results suggested that at a concentration higher than 5.1 µM quercetin-loaded PLGA NPs and EGCG may have cytotoxicity to the HCE cells. Therefore, we selected 2.55, 3.83, and 5.10 µM of quercetin in PLGA NPs and EGCG to further investigate the intracellular antioxidant activity.

### 2.8. Effects of the Quercetin-Loaded PLGA NPs Combined with EGCG on Intracellular ROS Levels

**Figure 9:**
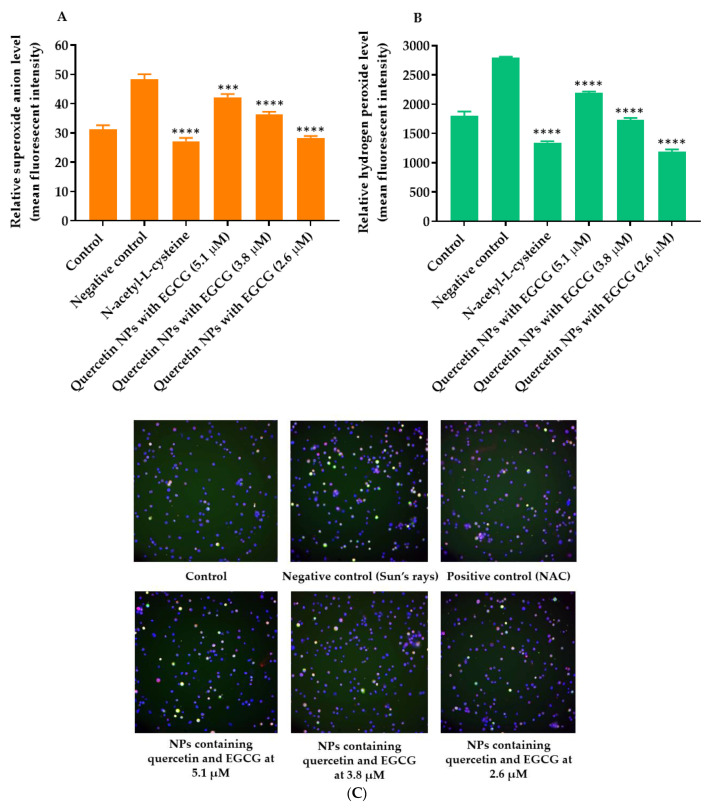
Effect of quercetin loaded PLGA NPs combined with EGCG on intracellular ROS generation. (**A**) Quantification of the hydrogen peroxide levels within the indicated groups. (**B**) Quantification of the superoxide anion levels within the indicated groups. (**C**) Fluorescence microscopy of the HCE cells treated with fluorescent probes after cultivation in the presence and absence of simulated-Sun-ray oxidative stress and the treatment with quercetin loaded PLGA NPs and EGCG. *** and **** indicate *p* < 0.001 and 0.0001 compared with control, respectively.

The cellular ROS generation was induced by applying simulated Sun’s rays to the HCE cell line. The results showed that hydrogen peroxide and superoxide anion levels in HCE cells that were exposed to the simulated Sun’s rays (negative control) increased significantly compared with those of untreated HCE cells. After the treatment with the positive control (5 mM n-acetyl-L-cysteine), the hydrogen peroxide and superoxide anion levels in HCE cells decreased significantly by up to 51.77 ± 0.64% and 43.74 ± 3.07%, respectively (Figure 9). The quercetin-loaded PLGA NPs combined with EGCG containing quercetin and EGCG at 5.1, 3.8, and 2.6 µM reduced the hydrogen peroxide levels by up to 22.01 ± 0.98, 37.60 ± 0.71, and 57.27 ± 1.15%, respectively, and reduced the superoxide anion levels by up to 12.76 ± 2.78, 24.71 ± 3.35, and 41.28 ± 1.97%, respectively. The results indicated that quercetin-loaded PLGA NPs combined with EGCG at a lower concentration (2.6 µM) had a comparative intracellular ROS inhibition compared with n-acetyl-L-cysteine (5 mM).

### 2.9. Gelation Temperature, Gelation Time, and pH of the Quercetin-Loaded PLGA NPs and EGCG Thermosensitive Gel Loaded In Situ

The pH values of the in situ thermosensitive gel base at 25 °C and 33 °C were 6.85 ± 0.01 and 6.76 ± 0.01, respectively. After being loaded with the quercetin-loaded PLGA NPs and EGCG, the pH values of the in situ gel at 25 °C and 33 °C were 6.69 ± 0.01 and 6.70 ± 0.01, respectively. These results indicated that the quercetin-loaded PLGA NPs and EGCG did not significantly change the pH of the thermosensitive gel, and the pH values were suitable for ocular delivery.

Both the in situ thermosensitive gel base and quercetin-loaded PLGA NPs and EGCG thermosensitive gel were liquids at 25 °C. The thermosensitive gel base formed a high-strength gel at 35 ± 0 °C, while the quercetin-loaded PLGA NPs and EGCG thermosensitive gel demonstrated a gelling temperature of 33 ± 0 °C (Table 1). The gelation time for the thermosensitive gel base and quercetin-loaded PLGA NPs and EGCG loaded in the gel were 300 and 157 ± 5.13 s, respectively.

### 2.10. Rheology of In Situ Thermosensitive Gel Loaded With Quercetin-Loaded PLGA NPs and EGCG

**Figure 10:**
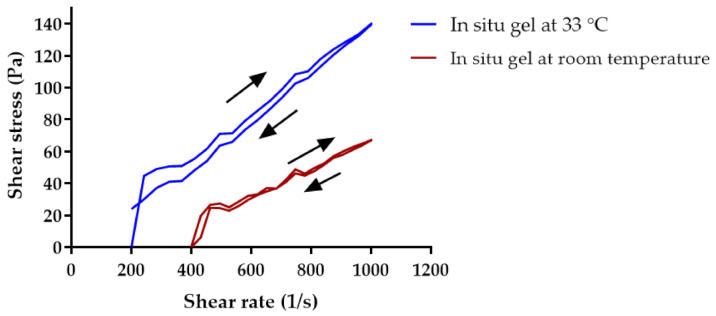
Rheology of the in situ gel loaded with quercetin-loaded PLGA NPs and EGCG at 33 °C and room temperature.

The shear stress and shear rate were plotted to evaluate the rheological behavior of the in situ thermosensitive gel loaded with quercetin-loaded PLGA NPs and EGCG. The thermosensitive gel had a decreased viscosity when the shear rate increased (Figure 10). However, it regained the viscosity as the shear rate decreased. These results suggested that the in situ thermosensitive gel loaded with quercetin-loaded PLGA NPs and EGCG had pseudoplastic and thixotropic types of rheology. Under the application of shearing, cross-linking between poloxamer chains was temporarily broken. This resulted in an increase in the fluidity of the gel, or the gel became a sol. When the shear rate decreased, the sol became semisolid (gel) again. The thixotropy property of the in situ gel was shown by the hysteresis loop, which meant that the gel took some time to return to a more viscous state. This phenomenon was observed because polymer chains and nanoparticles required time to reorganize [27].

## 3. Discussion

The cornea is a tissue that covers the front surface of the eye. Oxidation in the cornea is typically induced by UV radiation and oxidative stress. The human cornea absorbs 92% of sunlight, radiation, and oxygen [28]. Therefore, the cornea is always exposed to oxidative stress due to ROS, i.e., superoxide, hydroxyl, peroxyl, and hydroperoxyl radical accumulation. Normally, the cornea has a self-developed antioxidant defense mechanism containing non-enzymatic and enzymatic antioxidants. However, with aging, impaired antioxidant defense systems can cause the pathophysiological function of the cornea due to an imbalance between ROS production and clearance. ROS accumulation can result in DNA and cellular damage.

Quercetin is a natural enzymatic antioxidant. The antioxidant activity of quercetin has been extensively studied in recent years. The quercetin glutathione synthesis regulated the enzyme-mediated antioxidant system and regulated signal transduction pathways of reactive oxygen species. A DPPH assay was used to investigate the antioxidant effects of pure quercetin, pure loaded PLGA EGCG, quercetin loaded PLGA NPs, and the combination of quercetin-loaded PLGA NPs and EGCG. The combination of quercetin-loaded PLGA NPs and EGCG significantly enhanced the DPPH free radical scavenging. Interestingly, several reports showed that the combination of quercetin with catechin, epicatechin, or more than two catechin-related compounds resulted in antagonistic antioxidant activity due to their phenolic interaction during oxidation [29,30].

Quercetin was prone to oxidative degradation. Encapsulating quercetin in the PLGA NPs was undertaken to prolong the stability, control the release, and increase the solubility of quercetin. Hyaluronic acid adsorbed on the surface of quercetin-loaded PLGA NPs served multiple functions, including a stabilizer and a mucoadhesive polymer. HA is a glycosaminoglycan possessing a negative charge and high viscosity that could facilitate the adhesion of NPs to the ocular surface. It is one of the compositions of the corneal matrix that is biodegradable, biocompatible, non-irritating, and non-toxic. The mucoadhesive property of HA may increase the retention of quercetin-loaded PLGA NPs to the cornea. In this study, 0.1% HA significantly enhanced the colloidal stability of the quercetin-loaded PLGA NPs due to electrostatic and steric stabilization. The release of the quercetin from PLGA NPs had two different phases: a quick release of quercetin due to the burst release followed by a sustained release at a constant release rate. The release mechanisms were mainly based on the combination of drug diffusion, polymer swelling, and nanoparticle matrix erosion [31]. Regarding the stability study, quercetin-loaded PLGA NPs were stable in PBS, suggesting that any aggregation of quercetin-loaded PLGA NPs may not be observed upon instillation into the eyes. Quercetin was reported to degrade via photodegradation and oxidation [32]. In an aqueous medium, the quercetin in the PLGA matrix was found to decompose via a second-order reaction in which the reaction may occur between quercetin and oxygen. Wang and Zhao reported that the degradation rate constant of pure quercetin was increased at alkaline pH and higher temperatures [33].

The reduction in HCE cell viability after exposure to quercetin-loaded PLGA NPs was dose-dependent. The IC_50_ of quercetin to reduce the viability of breast cancer cells was reported at 50 µM, which agrees with our finding [34]. DCFH was a fluorescent dye that measured hydrogen and superoxide reactive oxygen species based on the diffusion of the probe into the cells. In the cytoplasm, the dye reacted with esterase enzymes to a non-fluorescent compound called trapped CM-DCFH, which reacted with ROS to form highly fluorescent products. The anti-cellular ROS study revealed the ability of quercetin loaded in PLGA NPs and EGCG at the non-cytotoxic concentrations to reduce the amount of ROS in HCE cells after exposure to the simulated Sun’s radiation. The results agreed with a previous study reporting that quercetin decreased UV-B-induced ROS production by HCE cells at 0.5 µM, although the ROS inhibition of quercetin NPs was never reported. The cellular ROS prevention of quercetin-loaded in PLGA NPs and EGCG decreased when the dose of quercetin loaded in PLGA NPs plus EGCG was increased. This might have been due to the increased cytotoxicity of quercetin-loaded PLGA NPs combined with EGCG. It was suggested that 2.6 µM of quercetin in NPs and EGCG inhibited ROS production by HCE cells [35].

An in situ thermosensitive gel containing quercetin-loaded PLGA NPs and EGCG was formulated. The in situ thermosensitive gel was designed to be a solution at room temperature to ease instillation and prevent blurred vision. After reaching the cornea, it can be retained at the ocular surface for a longer duration, thus increasing the residence time of quercetin-loaded PLGA NPs and EGCG, which may result in enhanced bioavailability of the active compounds. The sol-to-gel temperature observed in this developed in situ gel formulation was at 33 °C, which was suitable for ocular drug delivery. The literature indicated a normal ocular surface temperature range from 32.9 °C to 36 °C [36,37]. The viscosity of the gel increased with an increase in temperature. Loading the quercetin-loaded PLGA NPs and EGCG into the in situ thermosensitive gel at a physiological temperature significantly reduced the gelation time to 2.6 min, which was optimal for use.

## 4. Materials and Methods

### Materials

Quercetin, epigallocatechin gallate (EGCG), poloxamer 407, and hyaluronic acid sodium salt (HA) with Mws of 8000–15,000 were purchased from Myskinrecipe (Bangkok, Thailand). Poly(DL-lactic-co-glycolic acid) (50:50) with a terminal carboxyl group (PLGA, inherent viscosity 0.22 dL/g, molecular weight of 6700 Da) was purchased from Lactel Absorbable Polymers (Birmingham, AL, USA). MTT was purchased from Roche Applied Science (Penzberg, Germany). Keratinocyte serum-free medium (SFM), insulin, human recombinant zinc solution, collagen I bovine, human fibronectin, Dulbecco’s phosphate-buffered saline (PBS), fetal bovine serum (FBS), penicillin-streptomycin, trypan blue stain 0.4%, 0.25% trypsin-EDTA, and fluorobrite DMEM were purchased from Gibco (Waltham, MA, USA). The HCE cell line, supplements for keratinocyte consisting of epidermal growth factor (EGF), and bovine pituitary extract (BPE) were purchased from ATCC (Manassas, VA, USA)). Hydrocortisone solution and DPPH (2,2-diphenyl-1-picrylhydrazyl) were purchased from Sigma-Aldrich (St. Louis, MO, USA)). ROS/superoxide reagent kit was purchased from Abcam (Waltham, MA, USA).

## 5. Methods

### 5.1. Preparation of the Quercetin-Loaded PLGA NPs

The solvent displacement technique was used to prepare the quercetin-loaded PLGA NPs [38]. The quercetin-loaded PLGA NPs’ characteristics were optimized by varying the type and concentration of the stabilizer. PLGA (62.5 mg) and quercetin (6.25 mg) were completely dissolved in 5 mL of acetone. The solution (2 mL) was slowly introduced dropwise into 25 mL of aqueous phase containing 0.05%, 0.1%, and 0.2% *w*/*v* hyaluronic acid or 0.1% poloxamer at a rate of 8 mL/h under magnetic stirring (550 rpm). The nanoparticles were continuously stirred for 2 h to remove residual acetone and were kept in deionized water until characterization. The PLGA nanoparticles without quercetin were prepared for comparative studies.

### 5.2. Physical Characterization and Stability of the Quercetin-Loaded PLGA NPs

The size, size distribution, and surface charges of the quercetin-loaded PLGA NPs were characterized, and the physical stability of the NPs was evaluated. The hydrodynamic diameter, polydispersity index, and zeta potential values of the NPs were determined using a Zetasizer Nano ZS (Malvern Instruments, Worcestershire, UK) based on a dynamic light scattering technique and electrophoretic mobility, respectively [39]. Quercetin–PLGA NPs in deionized water (1.1 mg/mL) were poured into the light-protected vial and stored at 4 °C, 30 °C, and 45 °C for 12 weeks. At predetermined time intervals—0, 2, 4, 8, and 12 weeks—the sizes, PDIs, and zeta potentials of the NPs were determined.

### 5.3. Colloidal Stability of the Quercetin-Loaded PLGA NPs in Dulbecco’s PBS

PBS, pH 7.4, was used as a simulated biological fluid in the eyes as it has the pH and osmolality of normal tears under normal conditions [40]. The stability of the quercetin-loaded PLGA NPs dispersed in PBS was estimated by measuring the size and polydispersity index. The quercetin-loaded PLGA NPs were suspended in PBS, pH 7.4, and stored at 37 °C for 7 days. At a specific time, samples were taken for measuring the size and polydispersity index.

### 5.4. Encapsulation Efficiency and Release of the Quercetin from Quercetin-Loaded PLGA NPs

The encapsulation efficiency of quercetin in PLGA NPs was determined using a UV-visible spectrophotometry microplate reader (Molecular Devices, San Jose, CA, USA). The quercetin-loaded PLGA NPs (1.1 mg/mL) were centrifuged at 15,308× *g* for 10 min. Then, the supernatant was removed and the NPs were dissolved in DMSO. The absorbance of quercetin loaded in the NPs was measured at a λ_max_ of 380 nm. The calibration curve was plotted to display the absorbance as a function of quercetin concentration in a range of 1.22–156.25 µg/mL, with the R^2^ value of 0.9996, without the interference of PLGA and HA absorbance. The encapsulation efficiency and drug loading were calculated using Equations (1) and (2), respectively.

(1) $$\mathrm{Encapsulation}\;\mathrm{efficiency}\;\left(\%\right)=\frac{\mathrm{Amount}\;\mathrm{of}\;\mathrm{quercetin}\;\mathrm{added}-\mathrm{Amount}\;\mathrm{of}\;\mathrm{quercetin}\;\mathrm{in}\;\mathrm{NPs}}{\mathrm{Amount}\;\mathrm{of}\;\mathrm{quercetin}\;\mathrm{added}}\times 100$$

(2) $$\mathrm{Loading}\;\mathrm{efficiency}\;\left(\%\right)=\frac{\mathrm{Amount}\;\mathrm{of}\;\mathrm{quercetin}\;\mathrm{added}-\mathrm{Amount}\;\mathrm{of}\;\mathrm{quercetin}\;\mathrm{in}\;\mathrm{NPs}}{\mathrm{Amount}\;\mathrm{of}\;\mathrm{PLGA}}\times 100$$

The quercetin release profile from quercetin-loaded PLGA NPs was studied by incubating the nanoparticles in the PBS. The quercetin-loaded PLGA NPs was dispersed in PBS, pH 7.4, at a concentration of 0.55 mg/mL (equivalent to 0.24 mg quercetin) and incubated at 37 °C for 7 days. At selected time intervals, samples containing quercetin-loaded PLGA NPs were collected and centrifuged at 15,308× *g* for 10 min. The nanoparticles were collected and dissolved in DMSO. The amount of quercetin in the NPs was determined by measuring the absorbance at a λ_max_ of 380 nm using a UV-visible spectrophotometry microplate reader (Spectramax M3, Molecular Devices, San Jose, CA, USA). The amount of quercetin released from the quercetin-loaded PLGA NPs was calculated using a calibration curve. The cumulative release of quercetin (%) was calculated as per the Equation (3):

(3) $$\mathrm{Cumulative}\;\mathrm{release}\;\mathrm{of}\;\mathrm{quercetin}\;\left(\%\right)\;=\frac{\mathrm{Initial}\;\mathrm{amount}\;\mathrm{of}\;\mathrm{quercetin}\;\mathrm{in}\;\mathrm{NPs}-\mathrm{Amount}\;\mathrm{of}\;\mathrm{quercetin}\;\mathrm{remaining}\;\mathrm{in}\;\mathrm{NPs}\;}{\mathrm{Initial}\;\mathrm{amount}\;\mathrm{of}\;\mathrm{quercetin}\;\mathrm{in}\;\mathrm{NPs}}\times 100$$

### 5.5. Chemical Stability of the Quercetin-Loaded PLGA NPs

The chemical stability of the quercetin-loaded PLGA NPs was examined in deionized water. The quercetin-loaded PLGA NPs were stored at 4 °C, 30 °C, and 45 °C for 8 weeks. At fixed time intervals, the samples were collected and centrifuged at 15,308× *g* for 10 min. The supernatant was discarded and the quercetin-loaded PLGA NPs were dissolved in DMSO. The remaining amount of quercetin loaded in PLGA NPs was quantified using a UV-visible spectrophotometry microplate reader (Spectramax M3, Molecular Devices, San Jose, CA, USA) at a λ_max_ of 380 nm. The data regarding quercetin degradation were fitted with different kinetic equations, including zero-, first-, and second-order kinetics. The best-fitting model was identified using a statistical analysis. It was noted that the selection of the kinetic model was based on the goodness of fit and not based on the mechanism of the degradation of the complex system.

### 5.6. Antioxidant Activity of the Quercetin-Loaded PLGA NPs Mixed with EGCG

The in vitro antioxidant activity of the quercetin-loaded PLGA NPs was determined by using a DPPH free radical scavenging assay. Pure quercetin (1.95–1000 µg/mL), pure EGCG (1.95–1000 µg/mL), quercetin-loaded PLGA NPs and EGCG (equivalent to 0.18–94 µg/mL of quercetin and 0.2–100 µg/mL of EGCG), quercetin-loaded PLGA NPs (equivalent to 0.18–94 µg/mL), and blank PLGA NPs (0.2–1000 µg/mL) were dissolved or suspended in deionized water. All samples (100 µL) were mixed with 500 µM DPPH (100 µL) in absolute ethanol in 96-well plates. The mixtures were incubated in the dark at room temperature for 30 min. The absorbance was determined at 517 nm using a UV-visible microplate reader (Spectramax M3, Molecular Devices, San Jose, CA, USA). Absolute ethanol was used as a negative control. The percentage of radical scavenging activity was calculated using Equation (4):

(4) $$\mathrm{DPPH}\;\mathrm{radical}\;\mathrm{scavenging}\;\left(\%\right)=\frac{\left[A517\mathrm{control}-A517\mathrm{samples}\right]}{A517\mathrm{control}}\times 100$$

The sample concentration that decreased the DPPH free radicals to 50% of the initial concentration (IC_50_) was calculated using GraphPad Prism 7.

### 5.7. Cell Culture

The cell culture flask was coated with collagen I, bovine fibronectin, and bovine serum albumin for 20 min. A human corneal epithelial (HCE) cell line was cultured in keratinocyte serum-free medium (SFM) supplemented with 1% penicillin-streptomycin, 5 ng/mL epidermal growth factor (EGF), 0.05 mg/mL bovine pituitary extract (BPE), 500 ng/mL hydrocortisone, and 0.005 mg/mL insulin and maintained at 37 °C in a humidified incubator containing an atmosphere of 5% CO_2_.

### 5.8. Effects of the Quercetin-Loaded PLGA NPs on HCE Cell Viability

Quercetin (0.094, 0.02, 0.094, 0.94, 1.88, 9.4, 18.8, 47, 94 µg/mL) in PLGA NPs and EGCG (0.01, 0.02, 0.1, 1, 2, 10, 20, 50, 100 µg/mL) were diluted in the cell culture medium. HCE cells (3 × 10^4^ cells/well) were plated in 96-well plates and incubated for 72 h prior to the test. The medium was then removed and cells were rinsed one time with PBS. Then, the quercetin-loaded PLGA NPs combined with EGCG (200 µL) at various concentrations were added to the cells and incubated at 37 °C in 5% CO_2_ for 24 h. After incubation, the quercetin-loaded PLGA NPs and EGCG were removed and the cells were washed with PBS. MTT (1 mg/mL) was added to the cells (100 µL/well) and incubated with the cells for 4 h at 37 °C in 5% CO_2_. The MTT solution was removed and 100 μL of isopropanol was added to solubilize the water-insoluble formazan product. The absorbance was measured at 570 nm. The absorbance values were related to the number of viable cells. The cell viability percentage was calculated using Equation (5), where the control was the viability of untreated cells. The IC_50_ was calculated based on the non-linear regression analysis.

(5) $$\mathrm{Cell}\;\mathrm{viability}\;\left(\%\right)=\frac{A570\;\mathrm{of}\;\mathrm{tested}\;\mathrm{cells}}{A570\;\mathrm{of}\;\mathrm{control}}\times 100$$

The change in cellular morphology of the HCE cells was observed under an inverted microscope (EVOS, Thermo Fisher Scientific, Waltham, MA, USA). The score reactivity against the change of cellular morphology was given by the following criteria. Scores of 0, 1, 2, 3, and 4 indicated none, slightly toxic, mildly toxic, moderately toxic, and severely toxic reactivity, respectively.

### 5.9. Effects of the Quercetin-Loaded PLGA NPs Combined With EGCG on Intracellular ROS Levels

The quercetin-loaded PLGA NPs combined with EGCG at non-toxic concentrations were diluted in a keratinocyte serum-free medium. The HCE cells (1 × 10^5^ cells/mL) were plated in 24-well plates for 24 h at 37 °C in 5% CO_2_. The NPs and 5 mM N-acetyl-L-cysteine (ROS inhibitor, a positive control) were incubated with the cells for 30 min at 37 °C. After incubation, the samples were removed and the cells were washed with PBS (500 µL/well). Then, PBS (500 µL/well) was added to the cells before exposure to radiation that mimicked the Sun’s rays at a dose of 0.32 J/cm^2^ (OSRAM UV-lamp, Ultra-vitalux, Garching, Germany). The medium was removed and 0.25% trypsin-versene (300 µL) was added and incubated for 2 min to detach the cells. The cells were washed three times via centrifugation at 1200 rpm for 5 min at 4 °C. The green fluorescent dye (Ex/Em 490/525 nm; 1:2000), orange dye (Ex/Em 550/620 nm; 1:1000), and Hoechst 33,342 (5 µM) were incubated with the cells at 37 °C for 45 min. The cells were washed via centrifugation at 400× *g* for 5 min at room temperature. Then, fluorobrite DMEM was added to the cells. The cells were transferred to a 96-well plate to measure the fluorescent intensity by a fluorescence microplate reader (Tecan, Männedorf, Switzerland). The micrographs of cells were taken by fluorescence microscope (INCell analyzer, GE Health Care, Boston, MA, USA).

### 5.10. Preparation of Thermosensitive Gel Containing the Quercetin-Loaded PLGA NPs Combined with EGCG

The thermosensitive gel was prepared by dissolving 16 g of poloxamer 407 in 100 mL deionized water. The calculated amount of the quercetin-loaded PLGA NPs, and EGCG (equivalent to 0.94 µg/mL of quercetin and 1 µg/mL EGCG) were added to the poloxamer 407 solution under stirring (500 rpm). The mixture was stored at 4 °C until characterization.

### 5.11. Characterization of Thermosensitive Gel Containing the Quercetin-Loaded PLGA NPs Combined with EGCG

#### 5.11.1. Determination of Gelation Temperature

The mixture of poloxamer 407 and the quercetin-loaded PLGA NPs plus EGCG (3 mL) was poured in the glass tube and placed in a water bath. The initial temperature of water in the water bath was 28 °C and elevated in stepwise increments of 1 °C, which was measured by using a thermometer. The gelation temperature was assessed by tilting the tube 90° until the gel was immobilized. The sol-to-gel transition temperature was the lowest temperature when the gel was unable to mobilize.

#### 5.11.2. Determination of Gelation Time

The gelation time of thermosensitive gel containing the quercetin-loaded PLGA NPs and EGCG was assessed by filling the gel (3 mL) in the tube. The tube was immersed in the water bath where the water temperature was controlled at 33 ± 0.5 °C, which was a gelation temperature. The sol-to-gel transition time was determined by inverting the tube horizontally every minute. The time at which the gel was unable to mobilize was recorded as the gelation time.

#### 5.11.3. Rheological Measurement of Thermosensitive Gel Containing the Quercetin-Loaded PLGA NPs and EGCG

Measurement of the thermosensitive gel containing the quercetin-loaded PLGA NPs and EGCG rheology was performed using a rheometer (Brookfield) equipped with a plate and plate geometry. The ranging shear rate was from 200 to 1000 s^−1^ and 400 to 1000 s^−1^ for the thermosensitive gel at 33 °C and at room temperature, respectively. The thermosensitive gel was placed in a water bath to maintain the temperature at the gelation temperature.

#### 5.11.4. pH Measurement of the Thermosensitive Gel Loading Quercetin-Loaded PLGA NPs and EGCG

The pH of the thermosensitive gel containing the quercetin-loaded PLGA NPs and EGCG was measured in triplicates at 25 °C using a pH meter (Thermo Fisher Scientific, Waltham, MA, USA).

### 5.12. Statistical Analysis

Statistical analysis of data was completed using an analysis of variance (one-way ANOVA), followed by Newman–Keuls method as a post-hoc test to evaluate the significance of differences (GraphPad Prism 7.02, La Jolla, CA, USA). In all cases, a value of *p* < 0.05 was considered statistically significant. All data represent mean ± SD, *n* = 3 experiments.

## 6. Conclusions

Quercetin-loaded PLGA NPs were successfully developed and suitable for delivery of quercetin to human corneal epithelial cells. The encapsulation of quercetin into the NPs improved the quercetin solubility, controlled release, physical stability, and chemical stability. The combination of quercetin-loaded PLGA NPs and EGCG increased the antioxidant and intracellular ROS inhibition effects in HCE cells. Thus, quercetin-loaded PLGA NPs combined with EGCG may provide a new approach for delivering quercetin to reduce the reactive oxygen species in the eyes.

## Figures and Tables

**Table 1:** Mobility of the in situ thermosensitive gel containing quercetin-loaded PLGA NPs and EGCG upon increasing the temperature.

Temperature (°C)	In Situ Gel Base	In Situ Gel Containing Quercetin-Loaded PLGA NPs and EGCG
28	0 ± 0	1 ± 0
29	0 ± 0	1 ± 0
30	1 ± 0	1 ± 0
31	1 ± 0	1 ± 0
32	1 ± 0	2 ± 0
33	1 ± 0	2.7 ± 0.6
34	2 ± 0	3 ± 0
35	3 ± 0	3 ± 0
36	3 ± 0	3 ± 0
37	3 ± 0	3 ± 0
38	3 ± 0	3 ± 0
39	3 ± 0	0 ± 0
40	0 ± 0	0 ± 0

Score: 0 = freely mobilized, 1 = moderately mobilized, 2 = slightly mobilized, 3 = immobilized.

## Data Availability

Data is contained within the article.
